# Record of Meteorological Conditions at Buffalo, for Dec., 1855

**Published:** 1856-02

**Authors:** Sanford B. Hunt


					﻿ART. VI. — Record of Meteorological Conditions at Buffalo, for Dec.,
1855. By Sanford B. Hunt, M. D.
rB	•
ca	bos	a
«	b	g
be	3
B	*=
'Ss	3
S *	bo
03	B
b	b
o s	o
E	B
________________________________________________________________72	co_
•amjtuaduwx
;o aSu«j :jsd)Bd.i9	n	tn	m m in	nocix—ixco-o'^-o	m
. 'uoijB.dBAg jo	to . to	co co co .coco tn	ci
2	,(1™L	co	o' to to	to’ to nd nd cd cd cd to	—'	ad
-	.___________2?________CO	 Ct	Ct	CO CO CO	Cl	—_______________________________Ct	Cl
3	,1 'ajn,B C?	. co co co	co . .co to to	co io
-jodiuaj. uBop;	to	to	—	x <"	r-	x'	x to nd	cd -c’ o'	ci	ci	id
_______________25________co___-5	ci Cl____________d	ci	co co co	ci	—_______________________________ci	1 ca
1.	1	CO
a UOUB.dU.13 ......	O
2 jo	-duio r cd	—■’	<'■’ co’ -c’	to	to' o’ cd nd t-’	—<	no	ad t?	cd >
E	-t __________ co	co ci ci_____ co	ct co co co ci	ci	x---	ci ci
* fo	~ I	-	~
2 2, >.’aJnU<iuiaX| ^5	ci nd nd cd	ci ad to o’ ci ci ci nd	nd Z
E->	. ES	______L-**_________CO Cl Cl__________________________Cl co CO CO CO Cl — —1_______Cl Cl
2 ;
a ci 8-sL ?	M •	•	A-	u-^.
K ~	02	72	«j£	£	(S	JS|Z< 72 X
B	" O *	.	...	.	.	...................... ..
B	-5	—'	1- CO O	do	— OOCO-fd'S'CO	O -»■
p	--------------------------------------------------------------------------------
spno[3 joi.aiv|2	® coo 0	® co o m o 0 o 00 o o >
5 aoyB.dB.13 ...	....... .	......................no	In?
c io duia r. -redid o’x’nd o’ad x o’r-’cd cd o’nd x’ci o’nd x’ad ci ci-b-2 ad r^’io
g *	n jyj	COCO-O'-^'^CtCiClCOCOCOCl Cl Cl — ci — ct 'co
g	'3
g •ojry.daia.i,	o- _■	c; c; -x to’ o’ ci ci to cd ci 0 ci ci ci cd ' ci
B___________-t< CO	-f	-r-fCO-rcOCOCOCOCO-ai-l'-O'ClCO	CO -T	CO	CO	d	—	C	Cl	— Cl —	Ct 'CO
g _ 8	-d	^ .	.	CP p» Ed.......pC .	^	.	.	.	.& t?	jS
eg gO-S	72^	72 72	72X	72^72 72’72	72	E?	72	X
	 r- CO	_-i< C9	—	1.0	7>	09	09	G9C9	CC	CD 09	09	— CO	r-<	09	O'!	’T	»O	—	O?	70	
spno[^)jij )tca v |___________________________________________________________GO	~	***•	■~/	|
ul	J	1°
5 UOpU.dBA'J.............................. .	........................to t . CO
® io	dead l in in co od	in 09 ao cd cS cd cd ad	oo m* cri	—’	o o o‘oi o o ad-t Di r-
g _______^100^00 09.	CO Cg G9,^Q9,09 C9 09	G9	^ «r<	C0	CO CO G9 G9	r~f ~ <-i--g I09
s bl	~ S
.	>.	ainj.daiaj,	•	ad -t	o’ -i< o’ i-^	~	o’	o’ o’	o’	o’ nd o’	■<* t'	b- o’ cd	o’	ad o’	cd	nd o'	io
<1	SJ_____CO TC CO CO	CO CO	CO -T CO Cl	Cl	Ci	Cl -r	-o'	d Ci Cl	CO CO	CO Cl Cl	— Cl	' d
8^2. -d |£>' • .	.	• k.	.b^l
<	.°i	=	=S	«27QP^	7272	72 '72 ^	^^^727272^	«yj 72	.
1X1 o ?	.	.	..... .	.	... ...............
■ Cl CO -5 -5	Cl —	— r* no O	O	Cl	O O	Cl	Cl Cl r^	CO -r	Cl 01 O	CO	r- Cl	d	co -»	1
spnoiojoi.tnyjns ® nono oooxooooo 00 oouooooorotcooooO|
.	I ci -c COdO-fX-iClClCl Cl . Cl Cl CO 1O -b .00 d O Cl Cl x to Cl
•uio.iBqjo nil,-;	o-o--57j—• — pj pj ® o o’o o'o’o o’0 o’o’o’o o o cd ci o
b	;dCico?o“5o'aco do1 co TOCO COCO COCOCO CO COCO CO Cl CO CO CO CO CO Ql Cl col
<	'	o	' o 000	o	o
p	o	co—.oo	na	<0
” -uiBHJO-ui ct	no ci	T* •-!	t':	-1
'	_______’______________________________________________________I
---------"ainrri-* <M O of no to r- X O O — Cl CO —• m O b- x O C: — 01 co -r n-. — l- X ~ -Z — *
* Does not include snow, of which there was about 8 inches.
				

